# Motor performance in early life and participation in leisure‐time physical activity up to age 68 years

**DOI:** 10.1111/ppe.12467

**Published:** 2018-04-17

**Authors:** Ahmed Elhakeem, Rebecca Hardy, David Bann, Diana Kuh, Rachel Cooper

**Affiliations:** ^1^ MRC Unit for Lifelong Health and Ageing at UCL London UK; ^2^ MRC Integrative Epidemiology Unit at University of Bristol Bristol UK; ^3^ Centre for Longitudinal Studies UCL Institute of Education London UK

**Keywords:** exercise, life course, motor coordination, motor development, physical activity, sports

## Abstract

**Background:**

This study examined associations between motor performance in early life and leisure‐time physical activity (LTPA) participation across adulthood, and whether these changed with age.

**Methods:**

Data were from 2526 participants from the Medical Research Council National Survey of Health and Development. Motor indicators were mother‐reported ages at first standing and walking, teacher‐rated games ability at age 13, and finger‐ and foot‐tapping speed at age 15. LTPA was reported at ages 36, 43, 53, 60‐64, and 68 years and classed at each age as none, moderate (1‐4/mo), or regular (≥5/mo). Associations were examined using mixed‐effects Poisson regression models with robust error variance.

**Results:**

Better ability at games and faster tapping speed were associated with more frequent participation in LTPA across adulthood, for example, fully adjusted relative risk of regular LTPA across adulthood (vs none) for above‐average ability (vs below average or average) = 1.46 (95% CI 1.32, 1.61); and per 10‐unit higher number of finger‐taps/15 seconds = 1.04 (95% CI 1.02, 1.07). These associations did not vary by age (*P* ≥ .33 for interactions with age at LTPA). Ages at reaching motor milestones were not associated with LTPA (eg, fully adjusted relative risk of regular LTPA across adulthood for walking ≤10 and ≥18 months (vs 11‐17 months) were 1.01 (95% CI 0.86, 1.20) and 0.89 (95% CI 0.75, 1.06) respectively.

**Conclusions:**

Better ability at games and faster tapping speed in adolescence were associated with greater participation in LTPA throughout adult life; from age 36 up to age 68. Childhood motor skill interventions may therefore have the potential to promote lifelong LTPA.

## 1 INTRODUCTION

The many health benefits of leisure‐time physical activity (LTPA) include maintenance of physical capability,^1^ reduced risks of chronic diseases,^2^, ^3^ and attenuation of age‐related cognitive decline,^4^ all of which are important for ageing populations. Motor development that is, the process through which movement patterns and skills are acquired^5^ has also been related to adult health outcomes including muscle strength, fitness, and balance, which are known to be related to LTPA.^6^ For instance, in the MRC National Survey of Health and Development (NSHD),^7^ faster adolescent tapping speed, a neuropsychological indicator of motor speed and lateralised coordination,^8^ was related to better performance in chair rising and standing balance tests in midlife.^7^ In the 1966 Finnish birth cohort (NFBC66), earlier ages at standing and walking were related to higher muscle strength, endurance, and aerobic fitness at age 31^9^ while in the NSHD, earlier attainment of these motor milestones was associated with higher midlife grip strength.^10^ Therefore, it is plausible that early life motor function may influence LTPA in adulthood, for example, through the tracking of LTPA from childhood into adulthood (and with LTPA mediating associations with health outcomes like physical capability).

Children with higher levels of motor skill competence tend to participate more in sports and games during childhood^11^ and these associations have been shown to persist into adolescence.^11^, ^12^ Whether early life motor performance influences LTPA in and across adult life is unclear as only a few studies have examined these associations. For example, only one study has examined attainment of infant motor milestones in relation to adult LTPA.^13^ This study, which included Finnish twins, showed that, on average, the twin who began to stand and walk earlier (recalled around a decade later) reported higher LTPA at age 25 than their twin who reached these milestones later.^13^ In the 1970 British Birth Cohort (BCS), those with better ability to throw, balance, and walk backwards at age 10 were more likely to participate in LTPA at age 42^14^ and in the 1958 National Child Development Study (NCDS), teacher‐reports of the presence of hard coordination problems at age 7‐16 years were associated with nonparticipation in LTPA in mid‐adulthood.^15^

Most of these studies examined motor skills from later childhood and rely on a single measure of LTPA reported in earlier adulthood, and none have examined associations with LTPA in old age. Therefore, it remains unclear if associations extend across adult life up to older ages or whether they change with age. It is also unclear if different measures of motor performance, including those indicating the acquisition of the basic fundamental movement skills of standing and walking, development of more specialised and complex movements and object control skills, and those reflecting rapid information processing, are differentially associated with LTPA. Examining different markers of motor development from different ages in early life, and whether associations with LTPA change across life may both help identify underlying mechanisms.

The aim of this study was therefore to examine associations of all available prospectively ascertained markers of early‐life motor performance in the MRC NSHD; age at reaching infant motor milestones, ability at school games at age 13 and tests of finger‐ and foot‐tapping speed at age 15 years with levels of participation in LTPA across adulthood, and to investigate whether associations with LTPA change with age. We hypothesised that earlier attainment of milestones, better ability at games and faster tapping speed, as markers of better motor development, would be associated with greater participation in LTPA across adulthood between ages 36 and 68 years.

## 2 METHODS

### 2.1 Participants

The MRC NSHD is a nationally representative sample of 5362 British births during 1 week in March 1946 followed up regularly across life.^16^ At ages 36 (1982), 43 (1989), and 53 (1999) home‐based interviews were performed by trained nurses. At age 60‐64 (2006‐2010) study participants attended a clinical research facility or received a home visit and at age 68 (2014), a postal questionnaire was completed.

Of those contacted at ages 36 (n = 3322), 43 (n = 3262), 53 (n = 3035), 60‐64 (n = 2661), and 68 years (n = 2453) respectively, 99.6% (n = 3309), 100% (n = 3262), 98.5% (n = 2986), 82.2% (n = 2188), and 99.1% (n = 2431) provided information on LTPA, with a total of 3766 providing at least one measure (Figure S1). At ages 43, 53, and 60‐64, study participants were broadly representative of the native UK population born in the early post‐war years in terms of sociodemographic factors including sex, marital status, and social class.^17^, ^18^, ^19^ At the age 68 follow‐up, 84.2% (n = 2370) of the target sample, that is, those resident in the UK that were not deceased (957, 17.8%), previously withdrew consent (620, 11.6%), emigrated, and were no longer contactable (448, 8.3%) or untraceable for over 5 years (395, 7.4%) returned a postal questionnaire. An additional 83 study participants living abroad who remain in contact with the study also returned a questionnaire.^20^

Ethics approval was granted for each data collection; the most recent was approved by the Queen Square Research Ethics Committee (14/LO/1073) and the Scotland A Research Ethics Committee (14/SS/1009). All participants provided written informed consent.

### 2.2 Measurements

#### 2.2.1 Motor performance in early life

At age 2, mothers reported the infants’ ages at standing alone and walking several steps unsupported to the nearest month.^7^, ^10^ In addition to these continuous measures, ages at standing and walking were also categorised as early or late, approximately equivalent to 5th and 95th percentiles respectively, and compared with “average” developers.^21^

At age 13, in 1959, the school teacher who was most familiar with each study participant completed a school‐based questionnaire rating their ability in school games as above average, average or below average compared with their peers.^22^ No detail was collected on types of school games played however, in the 1950s, a new physical education curriculum was developed in British primary schools which promoted movement skills through activities including agility exercises with full apparatus (eg, ropes, bars, boxes), gymnastics, and movement to music.^23^, ^24^ For these analyses, those with below‐average and average ability at games were combined and compared to those with above‐average ability.

At age 15, finger and foot‐tapping speed tests were administered during a school‐based medical examination.^7^, ^10^ Finger‐tapping speed was assessed by recording the number of times in 15 seconds that participants could tap the dorsum of their right hand with the index finger of their left hand, the test was then repeated in reverse. Foot‐tapping speed was then assessed by recording the number of times in 15 seconds that participants could tap the ground with their left foot, the test was then repeated for the right foot. The fastest of the left and right index finger‐tapping scores and the left and right foot‐tapping scores were selected and grouped into multiples of 10 to derive two measures of motor speed/lateralised coordination: finger‐tapping and foot‐tapping.^7^, ^10^

#### 2.2.2 Leisure‐time physical activity across adulthood

At ages 36, 43, 53, 60‐64, and 68, study participants were asked to report how frequently they participated in LTPA at nurse interviews or using self‐completed questionnaires. At age 36, questions based on the Minnesota LTPA questionnaire were used to assess the number of times study members participated in 27 different sports, exercises, and other leisure activities during the previous month.^1^, ^2^, ^22^ At age 43, data were gathered on involvement in sports, exercise, or other vigorous leisure activities in the previous year including for how many months and how frequently in those months these activities were completed.^1^, ^2^ Finally, at ages 53, 60‐64, and 68, study members reported how frequently they took part in sports, exercise, or other vigorous leisure activities during the previous 4 weeks. For these analyses, at each age, study members were categorised as (i) inactive if they reported no participation in LTPA, (ii) moderately active if they took part up to 4 times, or (iii) regularly active if they participated 5 or more times (in the previous month at age 36, per month at age 43, and in the previous 4 weeks at ages 53, 60‐64, and 68). These categories allow for the comparable modelling of LTPA over 32 years and have previously been shown to consistently discriminate between individuals with different health prospects.^1^, ^2^, ^25^

#### 2.2.3 Confounding variables

Birthweight, birth order, childhood illness, and socio‐economic position were identified from the literature as potential confounders.^5^, ^6^, ^26^ Birthweight was extracted from birth records and birth order was reported by the mother. Information was obtained from mothers on serious childhood illness in the first 5 years of life which required a hospital stay lasting a minimum of 28 days.^22^ This information was used to group participants into whether they had any serious illness or not. Paternal occupational class at age 4 was used as a marker socio‐economic circumstances in childhood and grouped into 4 classes (I andII: professional, managerial or technical, IIINM: skilled non‐manual, IIIM: skilled manual, and IV andV: partly skilled or unskilled).^22^ Where paternal occupational class was missing (n = 173), values recorded at ages 11 and 15 were used instead. Chi‐squared tests confirmed that both these later measures were strongly associated with paternal occupational class at age 4; for example, of those with fathers in the highest occupational class at age 4, 88% remained in the same occupational class at age 11, and 86.1% at age 15.

### 2.3 Statistical analyses

Relative risks (RRs) of moderate (1‐4 times/mo) and regular (≥5 times/mo) LTPA across adulthood (vs no LTPA) by each motor indicator were estimated using mixed‐effects Poisson regression models with robust error variance.^27^ The mixed‐effects models account for dependence among repeated LTPA outcomes and allow all those with at least 1 of 5 repeated LTPA measures to be included under the missing at random assumption.^27^ Individuals with missing data on motor indicators and confounders were excluded from analyses. We also used these models to summarise the RRs of any LTPA (ie moderate or regular LTPA vs none) across adulthood. We examined whether probability of LTPA changes with age by testing indicator of motor development‐by‐age interaction terms.

Formal tests of sex interactions were carried out, and subsequent analyses were adjusted for sex after no evidence of interactions was found. Formal tests of deviation from the linear trend for milestones and tapping speed were also carried out and where evidence of non‐linear associations between milestones and LTPA were found subsequent models were run using categorised measures. All initial sex‐adjusted models were subsequently adjusted for birthweight, birth order, and childhood illness, and then for father's occupational class. To investigate if associations between adolescent tapping speed and LTPA were explained by the tracking of physical activity from early life, additional models were fitted with mutual adjustment for ability in school games as a proxy marker of childhood LTPA. Finally, we performed a sensitivity analysis to investigate the robustness of our estimates to unmeasured confounding by calculating the E‐values of our fully adjusted estimates.^28^ The E‐value [RR + sqrt(RR*(RR‐1)] represents the minimum strength of association that an unmeasured confounder would need to have with both exposure and outcome, conditional on measured confounders, to fully explain the observed association.^28^ For an RR of <1 we first took the inverse of the RR before applying the formula to calculate the E‐value. All analyses were carried out in STATA v15 (StataCorp, Texas).

## 3 RESULTS

### 3.1 Characteristics of study participants

In total, 2526 participants had complete data on ages at reaching infant motor milestones, ability in school games, finger‐ and foot‐tapping speed tests, and at least one measure of LTPA in addition to data on all selected confounders (Table 1, Figure S1). Of these, the majority had LTPA data from 4 of the 5 ages (a total of 9773 LTPA assessments between ages 36 and 68 were included in analyses). When compared with those excluded due to missing LTPA data, higher proportions of those with at least one measure of LTPA were female (49.6% vs 42.5%), had fathers in the highest occupational classes I andII (23.1% vs 20.9%) and were above average at games (19.0% vs 15.8%), and lower proportions had low birthweight (4.7% vs 8.8%) and serious childhood illness (6.5% vs 17.7%). At ages 36 and 43 years, higher proportions of men reported taking part in LTPA, but sex differences were less marked at older ages (Table 1). Earlier milestone attainment was related to better ability at school games but unrelated to finger‐and foot‐tapping speed, whereas above average games ability was associated with faster tapping speeds. Early attainment of milestones was more prevalent in those with lower paternal occupational class, whereas better games ability and faster tapping speed were associated with higher paternal occupational class.

**Table 1:** Characteristics of study participants with relevant data from the MRC NSHD, 1946‐2015

	Males, n = 1285 [n (%)]	Females, n = 1241 [n (%)]
Age at standing alone in months		
≤8	68 (5.3)	74 (6.0)
9‐14	1109 (86.3)	1087 (87.6)
≥15	108 (8.4)	80 (6.5)
Age at walking several steps unsupported in months		
≤10	101 (7.9)	103 (8.3)
11‐17	1081 (84.1)	1063 (85.7)
≥18	103 (8.0)	75 (6.0)
Ability in school games age 13 y		
Above average	251 (19.5)	233 (18.8)
Below average or average	1034 (80.5)	1008 (81.2)
Number of taps in 15 s at age 15 y (in multiples of 10)^a^		
Finger‐taps	5.8 (1.9)	5.5 (1.7)
Foot‐taps	5.1 (1.7)	5.0 (1.5)
Leisure‐time physical activity (LTPA) across adulthood: from ages 36 to 68 y		
LTPA age 36		
None	346 (30.2)	481 (42.6)
Moderate	306 (26.7)	277 (24.5)
Regular	495 (43.2)	371 (32.9)
LTPA age 43		
None	548 (48.7)	623 (57.0)
Moderate	269 (23.9)	245 (22.4)
Regular	308 (27.4)	225 (20.6)
LTPA age 53		
None	478 (47.6)	525 (50.2)
Moderate	190 (18.9)	176 (16.8)
Regular	336 (33.5)	344 (32.9)
LTPA age 60‐64		
None	498 (66.2)	494 (62.2)
Moderate	94 (12.5)	115 (14.5)
Regular	160 (21.3)	185 (23.3)
LTPA age 68		
None	485 (60.1)	520 (59.8)
Moderate	87 (10.8)	116 (13.3)
Regular	235 (29.1)	234 (26.9)
Birthweight (kg)		
≤2.50	44 (3.4)	64 (5.2)
2.51‐3.00	177 (13.8)	241 (19.4)
3.01‐3.50	429 (33.4)	470 (37.9)
3.51‐4.00	463 (36.0)	381 (30.7)
>4.00	172 (13.4)	85 (6.9)
Birth order		
First‐born	540 (42.0)	505 (40.7)
Second‐born	416 (32.4)	406 (32.7)
Third or later born	329 (25.6)	330 (26.6)
Serious childhood illness before age 5		
No	1197 (93.2)	1178 (94.9)
Yes	88 (6.9)	63 (5.1)
Father's occupational class age 4		
I andII: professional/managerial/technical	286 (22.3)	268 (21.6)
IIINM: skilled non‐manual	238 (18.5)	239 (19.3)
IIIM: skilled manual	401 (31.2)	377 (30.4)
IV andV: partly skilled or unskilled	360 (28.0)	357 (28.8)

MRC NSHD: Medical Research Council National Survey of Health and Development.

a Data for tapping speed shows mean and standard deviation. Moderate LTPA: 1‐4 times/mo. Regular LTPA: ≥5 times/mo.

### 3.2 Infant motor milestones and LTPA across adulthood

**Figure 1:**
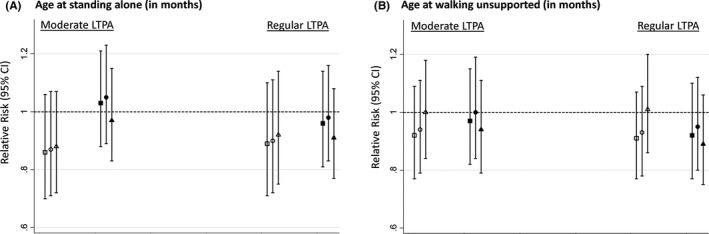
Relative risks (95% CI) of moderate (1‐4/mo) and regular (≥5/mo) participation in LTPA between ages 36 and 68 y (vs none) by age at reaching infant motor milestones. A, age at standing alone in months (hollow markers: ≤8, dark markers: ≥15, reference: ≥9‐14). B, age at walking several steps unsupported in months (hollow markers: ≤10, dark markers: ≥18, reference: 11‐17. Square: adjusted for sex. Circle: adjusted for sex, birth weight, birth order, and serious childhood illness. Triangle: as for circle plus adjustments for father's occupational class. Models include sex‐by‐age at LTPA assessment interaction term

There was little evidence that ages at standing and walking were associated with LTPA at either a moderate or regular level across adulthood (Figure 1). There was no evidence of an interaction between attainment of milestones and age at LTPA suggesting associations did not differ by age at assessment of LTPA (*P* = .22 and *P* = .60 respectively for age at standing‐ and age at walking‐by‐age at LTPA interactions). This is consistent with the similar RRs of LTPA at each age in adulthood (Table S1). The fully adjusted RRs of any LTPA between 36 and 68 years were 0.92 (95% CI 0.80, 1.05) for standing ≤8 months and 0.96 (95% CI 0.87, 1.06) for standing ≥ 15 months (vs average), and 0.99 (95% CI 0.90, 1.10) for walking ≤10 months and 0.94 (95% CI 0.84, 1.04) for walking ≥18 months (vs average).

### 3.3 Ability at school games at age 13 and LTPA across adulthood

**Figure 2:**
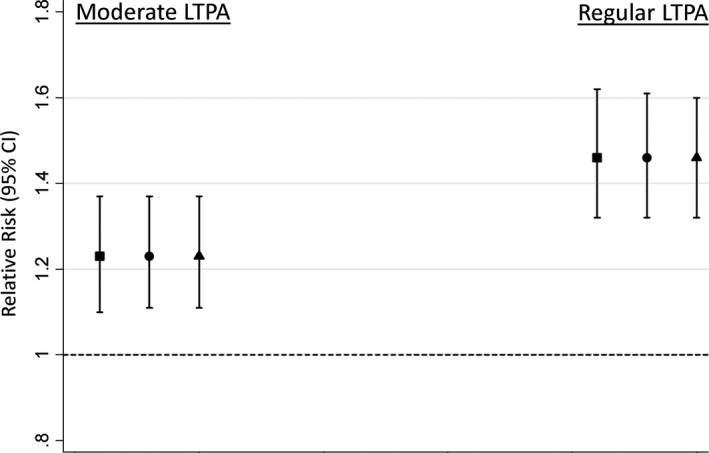
Relative risks (95% CI) of moderate (1‐4/mo) and regular (≥5/mo) participation in LTPA between ages 36 and 68 y (vs none) by teacher‐rated ability at school games. Estimates are for above‐average ability, reference = below‐average or average ability. Square: adjusted for sex. Circle: adjusted for sex, birth weight, birth order, and serious childhood illness. Triangle: as for circle plus adjustments for father's occupational class. Models include sex‐by‐age at LTPA assessment interaction term

Those rated by their school teacher as above average at games had higher likelihood of LTPA across adulthood when compared with those rated as below average or average (Figure 2). There was a trend such that above‐average ability was related to higher likelihood of more frequent LTPA (Figure 2). There was no evidence of an interaction between ability at games and age suggesting associations did not differ by age at assessment of LTPA (*P* = .74 for ability at games‐by‐age at LTPA interaction). This is consistent with the similar RRs of LTPA at each age in adulthood (Table S2). Similar associations were found after adjustment for covariates (Figure  2). The fully adjusted RR of any LTPA across adulthood for above average games ability was 1.24 (95% CI 1.17, 1.31).

### 3.4 Tapping speed at age 15 and LTPA across adulthood

**Figure 3:**
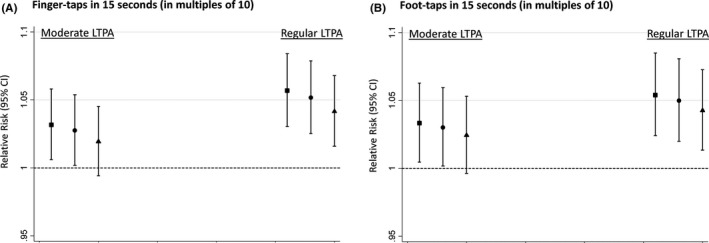
Relative risks (95% CI) of moderate (1‐4/mo) and regular (≥5/mo) participation in LTPA between ages 36 and 68 y (vs none) per 10‐unit higher number of A, finger and B, foot taps in 15 seconds. Square: adjusted for sex. Circle: adjusted for sex, birth weight, birth order, and serious childhood illness. Triangle: as for circle plus adjustments for father's occupational class. Models include sex‐by‐age at LTPA assessment interaction term

Faster finger‐ and foot‐tapping speeds were associated with higher likelihood of LTPA across adulthood (Figure 3). There was a trend such that faster tapping speed was related to higher likelihood of more frequent LTPA and estimates were only slightly attenuated after adjustment for covariates (Figure 3). These associations did not differ by age at assessment of LTPA (tapping speed‐by‐age at LTPA interaction terms: *P* = .33 for finger‐tapping and *P* = .48 for foot‐tapping, and Table S3). The fully adjusted RRs of any LTPA across adulthood per 10‐unit higher number of finger‐taps and foot‐taps in 15 seconds were 1.02 (95% CI 1.01, 1.04) and 1.03 (95% CI 1.01, 1.05) respectively.

Associations were maintained after mutual adjustment for ability in school games (using separate models for finger‐ and foot‐tapping), eg, RR of regular LTPA for above‐average ability and per 10‐unit higher number of finger taps were 1.45 (95% CI 1.32, 1.60) and 1.04 (95% CI 1.01, 1.07) respectively. Examining E‐values of our fully adjusted models suggest that unmeasured confounding of considerable strength would be needed to fully explain the observed associations (Table S4), for example, E‐values for RRs of regular LTPA were 2.28 for ability at school games and 1.24 for both finger‐ and foot‐tapping speed.

## 4 COMMENT

### 4.1 Principal findings

We examined how motor performance in early life relates to participation in LTPA from ages 36 to 68 years in the oldest of the British birth cohort studies. Above average ability at school games and faster finger‐ and foot‐tapping speeds in adolescence were associated with both higher likelihood and greater levels of participation in LTPA across adulthood. These associations did not vary by age and were robust to adjustment for measured confounders. There was limited evidence of associations between ages at reaching infant motor milestones and adulthood LTPA.

### 4.2 Interpretation

The finding that better ability at games and motor coordination in adolescence were associated with LTPA up to the sixth decade of life extends findings from NCDS showing that childhood hand coordination problems and lower self‐rated sports aptitude at 16 were related to lower odds of LTPA between ages 33 and 50.^15^ It also builds on findings from BCS70 of associations between poorer throwing, balance, and walking backwards skills at age 10 and lower likelihood of LTPA at age 42.^14^ Our findings extend earlier findings from NSHD showing that better school games ability was related to LTPA at age 36^22^ by showing that associations persist into old age. Conversely, our findings do not support associations reported between earlier attainment of motor milestones and higher sports participation at age 14 in NFBC66^29^ or findings that Finnish twins who learned to stand and walk earlier (as recalled by parents at age 11‐12 years) reported higher LTPA at age 25 than their less advanced twin.^13^

Our findings suggest that motor performance in adolescence, but not the timing of infant motor milestone attainment, may influence levels of participation in LTPA throughout adulthood including at older ages.^11^ Greater competence in movement and hand‐eye coordination skills in childhood may promote enjoyment and involvement in sports and help to establish an active life style, whereas less rewarding experiences during sport due to lower competence may discourage participation.^11^ Those with better motor skills and high competence in activities such as school team sports may continue to participate in LTPA into and across adulthood, consistent with the tracking of LTPA from childhood to adulthood.^30^ Finger‐ and foot‐tapping speeds have not previously been examined in relation to later LTPA. The findings for these psychomotor indicators may reflect differences in development of the brain and nervous systems since finger‐tapping is a marker of upper limb motor function including sensorimotor brain function^31^ and foot‐tapping is a marker of lower limb motor function controlled by the lateral descending system.^32^ This is supported by the fact that faster tapping speed was related to better balance in midlife in NSHD.^7^ The difference in findings between infant milestones and the adolescent indicators of motor performance may reflect differences between motor development in these 2 early life stages.

### 4.3 Strengths of the study

Loss to follow‐up in NSHD led to only small differences between included participants and those excluded due to missing data. The mixed‐effects models allowed all those with at least 1 of 5 repeated LTPA measures to be included, which helps to increase sample size thereby reducing bias due to missing outcome data, and subsequently improves precision of estimates.^27^ Furthermore, mixed‐effects models facilitated formal testing of whether associations of motor indicators with LTPA change with age. Other strengths include availability of LTPA data spanning 32 years of adulthood up to old age, examining associations with levels of LTPA by different and prospectively ascertained indicators of motor development that included previously unexamined and physician assessed markers of motor speed and lateralised coordination.

### 4.4 Limitations of the data

The LTPA data used were reported by study participants and thus recall and misclassification are possible, though these self‐reported data help to identify activities within a certain domain specifically leisure‐time.^33^ Additionally, when LTPA and monitor‐based activity measures were compared in a subsample of this cohort, those participating in LTPA across adulthood were found to spend greater time in higher intensity activity when compared with others reporting no LTPA,^34^ and these LTPA measures have also been shown to be associated with significant differences in adult health outcomes.^1^, ^2^, ^25^

Missing outcome data were included under the missing at random assumption (ie probability of missing data assumed to be related to measured factors but unrelated to unmeasured factors).Although it is not possible to test that the assumption of missing at random holds, we do not suspect that LTPA data were not missing at random. Missing data on motor indicators and confounders were excluded from the analyses which might introduce bias. Lastly, we did not have measures of early life LTPA and thus we were unable to assess if associations were due to the tracking of LTPA into adulthood. However, results of models adjusted for ability in school games suggest that associations between tapping speed and adult LTPA are unlikely to be fully explained by this.

## 5 CONCLUSIONS

Our findings suggest that interventions effective at improving motor skills of children and adolescents^35^ may promote greater participation in LTPA throughout adult life, though the associations reported here may not necessarily be causal. Future studies should integrate findings from different epidemiological approaches (eg cross‐cohort comparison and instrumental variable analysis) to help improve causal inference in the associations reported. In conclusion, in the 1946 British birth cohort, above‐average ability at games and faster finger‐ and foot‐tapping speed in adolescence were independently associated with greater levels of LTPA across adulthood up to age 68, whereas limited evidence was found relating ages at reaching infant motor milestones to adulthood LTPA.

## CONFLICT OF INTEREST

The authors declare that they have no competing interests.
