# Supplementary material for: Motor performance in early life and participation in leisure‐time physical activity up to age 68 years
Source: Paediatr Perinat Epidemiol. 2018 Apr 17;32(4):327–34. doi: 10.1111/ppe.12467 (PMC6099324; doi:10.1111/ppe.12467)
Supplement: Supplementary file 1 [file PPE-32-327-s001.pdf]

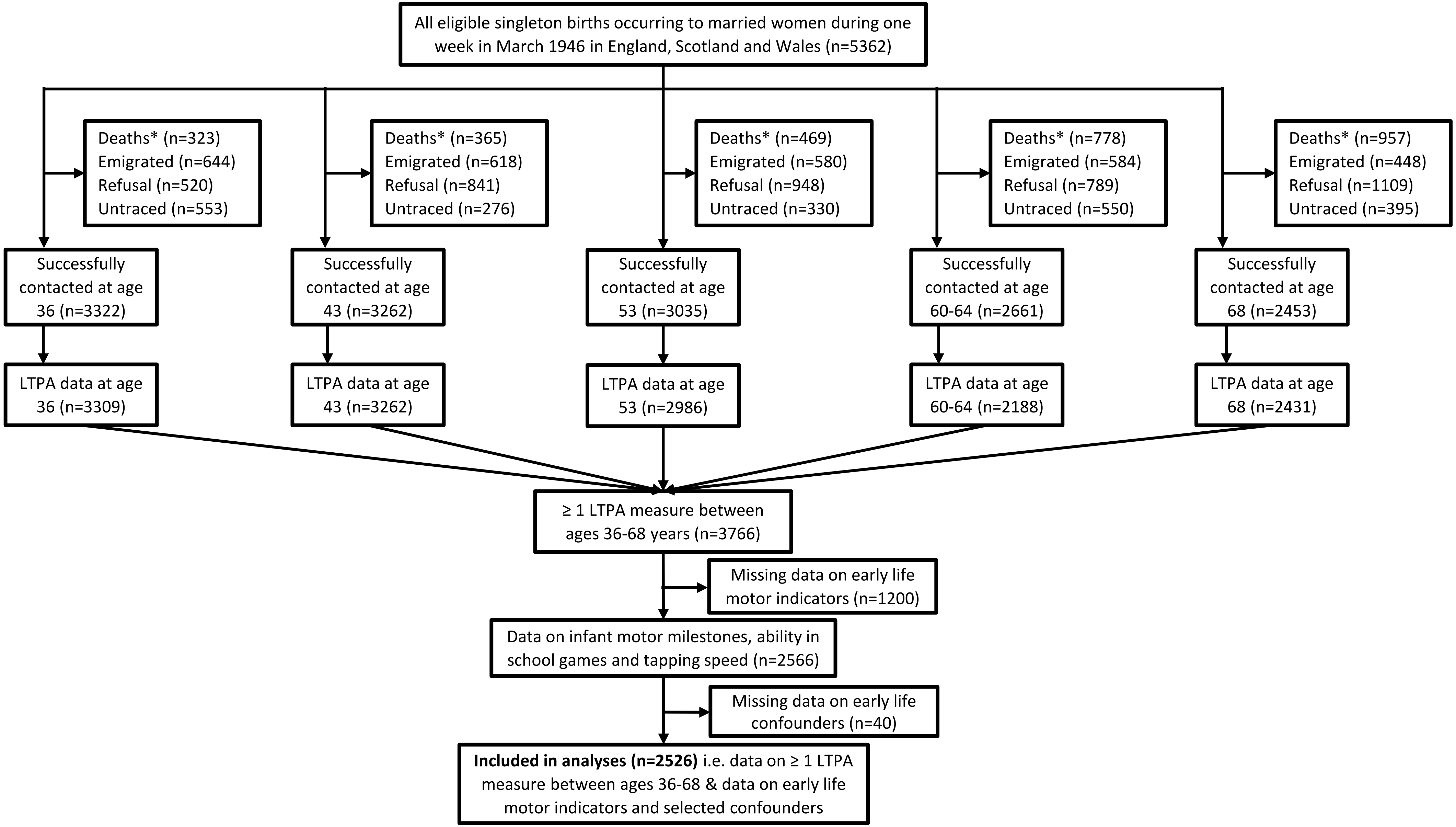

Note: Refusal includes temporary and permanent refusals and those that were in the target sample but did not respond at the specified age. Emigrated includes those temporarily living abroad.

* Number of deaths is cumulative
