# Supplementary material for: Motor performance in early life and participation in leisure‐time physical activity up to age 68 years
Source: Paediatr Perinat Epidemiol. 2018 Apr 17;32(4):327–34. doi: 10.1111/ppe.12467 (PMC6099324; doi:10.1111/ppe.12467)
Supplement: Supplementary file 2 [file PPE-32-327-s002.docx]

**eTable 1.** Age at reaching infant motor milestones (in months) and leisure-time physical activity (LTPA) at each age in adulthood, 1946-2015

| RR (95% CI) of participation in LTPA ≥ once per month | | | |  |
| --- | --- | --- | --- | --- |
|  | Model 1 | Model 2 | Model 3 | |
|  |  |  |  | |
| *LTPA age 36 years (n=2901)* |  |  |  | |
| *Standing* |  |  |  | |
| ≤ 8m (n=155) | 0.97 (0.86, 1.11) | 0.98 (0.86, 1.11) | 0.99 (0.88, 1.12) | |
| 9-14m (n=2511) | 1.00 (Reference) | 1.00 (Reference) | 1.00 (Reference) | |
| ≥ 15m (n=235) | 0.98 (0.89, 1.09) | 0.99 (0.90, 1.10) | 0.97 (0.87, 1.07) | |
| *Walking* |  |  |  | |
| ≤ 10m (n=243) | 0.94 (0.84, 1.04) | 0.94 (0.88, 1.10) | 0.97 (0.87, 1.08) | |
| 11-17m (n=2431) | 1.00 (Reference) | 1.00 (Reference) | 1.00 (Reference) | |
| ≥ 18m (n=227) | 1.00 (0.90, 1.10) | 1.02 (0.92, 1.13) | 0.99 (0.89, 1.10) | |
| *LTPA age 43 years (n=2848)* |  |  |  | |
| *Standing* |  |  |  | |
| ≤ 8m (n=148) | 0.93 (0.78, 1.12) | 0.94 (0.78, 1.13) | 0.96 (0.80, 1.16) | |
| 9-14m (n=2467) | 1.00 (Reference) | 1.00 (Reference) | 1.00 (Reference) | |
| ≥ 15m (n=233) | 1.01 (0.88, 1.16) | 1.03 (0.90, 1.19) | 0.98 (0.85, 1.12) | |
| *Walking* |  |  |  | |
| ≤ 10m (n=226) | 0.79 (0.67, 0.94) | 0.81 (0.68, 0.96) | 0.85 (0.72, 1.01) | |
| 11-17m (n=2397) | 1.00 (Reference) | 1.00 (Reference) | 1.00 (Reference) | |
| ≥ 18m (n=225) | 1.03 (0.90, 1.18) | 1.07 (0.94, 1.23) | 1.02 (0.89, 1.17) | |
| *LTPA age 53 years (n=2615)* |  |  |  | |
| Standing |  |  |  | |
| ≤ 8m (n=134) | 0.91 (0.75, 1.09) | 0.92 (0.76, 1.10) | 0.93 (0.77, 1.12) | |
| 9-14m (n=2268) | 1.00 (Reference) | 1.00 (Reference) | 1.00 (Reference) | |
| ≥ 15m (n=213) | 1.13 (1.00, 1.28) | 1.17 (1.04, 1.33) | 1.11 (0.98, 1.26) | |
| Walking |  |  |  | |
| ≤ 10m (n=208) | 0.92 (0.80, 1.08) | 0.94 (0.81, 1.09) | 0.99 (0.85, 1.15) | |
| 11-17m (n=2199) | 1.00 (Reference) | 1.00 (Reference) | 1.00 (Reference) | |
| ≥ 18m (n=208) | 1.00 (0.87, 1.14) | 1.04 (0.90, 1.19) | 0.98 (0.86, 1.13) | |
| *LTPA age 60-64 years (n=1920)* |  |  |  | |
| Standing |  |  |  | |
| ≤ 8m (n=92) | 0.85 (0.62, 1.16) | 0.85 (0.62, 1.16) | 0.85 (0.62, 1.17) | |
| 9-14m (n=1668) | 1.00 (Reference) | 1.00 (Reference) | 1.00 (Reference) | |
| ≥ 15m (n=160) | 1.00 (0.80, 1.24) | 1.03 (0.83, 1.29) | 0.97 (0.78, 1.21) | |
| Walking |  |  |  | |
| ≤ 10m (n=142) | 0.88 (0.69, 1.13) | 0.89 (0.69, 1.14) | 0.94 (0.73, 1.21) | |
| 11-17m (n=1625) | 1.00 (Reference) | 1.00 (Reference) | 1.00 (Reference) | |
| ≥ 18m (n=153) | 1.00 (0.80, 1.24) | 1.07 (0.86, 1.33) | 1.01 (0.81, 1.26) | |
| *LTPA age 68 years (n=2135)* |  |  |  | |
| Standing |  |  |  | |
| ≤ 8m (n=98) | 0.77 (0.58, 1.04) | 0.77 (0.58, 1.04) | 0.78 (0.58, 1.05) | |
| 9-14m (n=1859) | 1.00 (Reference) | 1.00 (Reference) | 1.00 (Reference) | |
| ≥ 15m (n=178) | 0.92 (0.76, 1.12) | 0.94 (0.77, 1.14) | 0.87 (0.71, 1.06) | |
| Walking |  |  |  | |
| ≤ 10m (n=144) | 0.83 (0.66, 1.05) | 0.83 (0.66, 1.05) | 0.88 (0.70, 1.12) | |
| 11-17m (n=1818) | 1.00 (Reference) | 1.00 (Reference) | 1.00 (Reference) | |
| ≥ 18m (n=173) | 0.92 (0.75, 1.12) | 0.94 (0.77, 1.15) | 0.89 (0.72, 1.08) | |

RR: Relative risk. 95CI: 95% confidence intervals. N=maximum sample size available at each adult age. Model 1: adjusted for sex. Model 2: adjusted for sex, birth weight, birth order and serious childhood illness. Model 3: model 2 plus adjustments for father’s occupational class. Models at age 60-64 were also adjusted for age.
