# Supplementary material for: Motor performance in early life and participation in leisure‐time physical activity up to age 68 years
Source: Paediatr Perinat Epidemiol. 2018 Apr 17;32(4):327–34. doi: 10.1111/ppe.12467 (PMC6099324; doi:10.1111/ppe.12467)
Supplement: Supplementary file 3 [file PPE-32-327-s003.docx]

**eTable 2**. Teacher-rated ability at school games at age 13 years and leisure-time physical activity (LTPA) at each age in adulthood, 1946-2015

| RR (95% CI) of participation in LTPA ≥ once per month | | | |
| --- | --- | --- | --- |
|  | Model 1 | Model 2 | Model 3 |
|  |  |  |  |
| *Ability at school games at age 13 years* |  |  |  |
| *LTPA age 36 years (n=2792)* |  |  |  |
| above average ability (n=532) | 1.16 (1.09, 1.23) | 1.15 (1.09, 1.23) | 1.15 (1.08, 1.23) |
| Below average or average ability (n=2260) | 1.00 (Reference) | 1.00 (Reference) | 1.00 (Reference) |
| *LTPA age 43 years (n=2848)* |  |  |  |
| above average ability (n=516) | 1.25 (1.15, 1.37) | 1.25 (1.14, 1.36) | 1.24 (1.14, 1.36) |
| Below average or average ability (n=2205) | 1.00 (Reference) | 1.00 (Reference) | 1.00 (Reference) |
| *LTPA age 53 years (n=2615)* |  |  |  |
| above average ability (n=456) | 1.18 (1.08, 1.29) | 1.17 (1.07, 1.28) | 1.17 (1.07, 1.28) |
| Below average or average ability (n=2039) | 1.00 (Reference) | 1.00 (Reference) | 1.00 (Reference) |
| *LTPA age 60-64 years (n=1920)* |  |  |  |
| above average ability (n=357) | 1.49 (1.31, 1.69) | 1.50 (1.32, 1.70) | 1.50 (1.32, 1.71) |
| Below average or average ability (n=1505) | 1.00 (Reference) | 1.00 (Reference) | 1.00 (Reference) |
| *LTPA age 68 years (n=2135)* |  |  |  |
| above average ability (n=399) | 1.27 (1.13, 1.43) | 1.27 (1.13, 1.44) | 1.28 (1.14, 1.44) |
| Below average or average ability (n=1651) | 1.00 (Reference) | 1.00 (Reference) | 1.00 (Reference) |

RR: Relative risk. 95CI: 95% confidence intervals. N=maximum sample size available at each adult age. Model 1: adjusted for sex. Model 2: adjusted for sex, birth weight, birth order and serious childhood illness. Model 3: model 2 plus adjustments for father’s occupational class. Models at age 60-64 were also adjusted for age.
