# Supplementary material for: Motor performance in early life and participation in leisure‐time physical activity up to age 68 years
Source: Paediatr Perinat Epidemiol. 2018 Apr 17;32(4):327–34. doi: 10.1111/ppe.12467 (PMC6099324; doi:10.1111/ppe.12467)
Supplement: Supplementary file 4 [file PPE-32-327-s004.docx]

**eTable 3.** Tapping speed at age 15 years and leisure-time physical activity (LTPA) at each age in adulthood, 1946-2015.

| RR (95% CI) of participation in LTPA ≥ once per month | | | |
| --- | --- | --- | --- |
|  | Model 1 | Model 2 | Model 3 |
|  |  |  |  |
| *Tapping speed (per 10-unit higher number of taps)* |  |  |  |
| *LTPA age 36 years (n=2592)* |  |  |  |
| finger-tapping | 1.02 (1.00, 1.04) | 1.02 (1.00, 1.03) | 1.01 (1.00, 1.03) |
| foot-tapping | 1.02 (1.00, 1.04) | 1.02 (1.00, 1.04) | 1.02 (1.00, 1.04) |
| *LTPA age 43 years (n=2530)* |  |  |  |
| finger-tapping | 1.04 (1.01, 1.05) | 1.03 (1.01, 1.06) | 1.02 (1.00, 1.05) |
| foot-tapping | 1.04 (1.02, 1.07) | 1.04 (1.01, 1.06) | 1.03 (1.01, 1.06) |
| *LTPA age 53 years (n=2322)* |  |  |  |
| finger-tapping | 1.03 (1.00, 1.05) | 1.02 (1.00, 1.05) | 1.02 (0.99, 1.04) |
| foot-tapping | 1.02 (1.00, 1.05) | 1.02 (1.00, 1.05) | 1.01 (0.99, 1.04) |
| *LTPA age 60-64 years (n=1736)* |  |  |  |
| finger-tapping | 1.06 (1.03, 1.10) | 1.06 (1.02, 1.10) | 1.05 (1.01, 1.09) |
| foot-tapping | 1.07 (1.03, 1.12) | 1.07 (1.03, 1.12) | 1.07 (1.02, 1.11) |
| *LTPA age 68 years (n=1900)* |  |  |  |
| finger-tapping | 1.05 (1.02, 1.08) | 1.05 (1.02, 1.08) | 1.04 (1.01, 1.07) |
| foot-tapping | 1.05 (1.02, 1.09) | 1.05 (1.02, 1.09) | 1.05 (1.01, 1.08) |

RR: Relative risk. 95CI: 95% confidence intervals. N=maximum sample size available at each adult age. Model 1: adjusted for sex. Model 2: adjusted for sex, birth weight, birth order and serious childhood illness. Model 3: model 2 plus adjustments for father’s occupational class. Models at age 60-64 were also adjusted for age.
