# Supplementary material for: Motor performance in early life and participation in leisure‐time physical activity up to age 68 years
Source: Paediatr Perinat Epidemiol. 2018 Apr 17;32(4):327–34. doi: 10.1111/ppe.12467 (PMC6099324; doi:10.1111/ppe.12467)
Supplement: Supplementary file 5 [file PPE-32-327-s005.docx]

|  | Regular LTPA | Moderate LTPA | Any LTPA |
| --- | --- | --- | --- |
| Ability at school games | 2.28 | 1.76 | 1.79 |
| Finger-tapping speed | 1.24 | 1.16 | 1.16 |
| Foot-tapping speed | 1.24 | 1.16 | 1.21 |

**eTable 3** E-values (in relative risk (RR) scale) of confounder-adjusted RRs

The E-value [RR+sqrt(RR*(RR-1)] represents the minimum strength of association that an unmeasured confounder would need to have with both exposure and outcome, conditional on measured confounders, to fully explain the observed association.^(28)^
